# Harnessing Natural Language Processing to Support Decisions Around Workplace-Based Assessment: Machine Learning Study of Competency-Based Medical Education

**DOI:** 10.2196/30537

**Published:** 2022-05-27

**Authors:** Yusuf Yilmaz, Alma Jurado Nunez, Ali Ariaeinejad, Mark Lee, Jonathan Sherbino, Teresa M Chan

**Affiliations:** 1 McMaster Education Research, Innovation, and Theory Program Faculty of Health Sciences McMaster University Hamilton, ON Canada; 2 Department of Medical Education Ege University Izmir Turkey; 3 Program for Faculty Development Office of Continuing Professional Development McMaster University Hamilton, ON Canada; 4 Department of Medicine Faculty of Health Sciences McMaster University Hamilton, ON Canada; 5 Department of Medicine and Masters in eHealth Program McMaster University Hamilton, ON Canada; 6 Division of Emergency Medicine, Department of Medicine Faculty of Health Sciences McMaster University Hamilton, ON Canada; 7 Division of Education and Innovation, Department of Medicine Faculty of Health Sciences McMaster University Hamilton, ON Canada

**Keywords:** natural language processing, machine learning algorithms, competency-based medical education, assessment, medical education, medical residents, machine learning, work performance, prediction models

## Abstract

**Background:**

Residents receive a numeric performance rating (eg, 1-7 scoring scale) along with a narrative (ie, qualitative) feedback based on their performance in each workplace-based assessment (WBA). Aggregated qualitative data from WBA can be overwhelming to process and fairly adjudicate as part of a global decision about learner competence. Current approaches with qualitative data require a human rater to maintain attention and appropriately weigh various data inputs within the constraints of working memory before rendering a global judgment of performance.

**Objective:**

This study explores natural language processing (NLP) and machine learning (ML) applications for identifying trainees at risk using a large WBA narrative comment data set associated with numerical ratings.

**Methods:**

NLP was performed retrospectively on a complete data set of narrative comments (ie, text-based feedback to residents based on their performance on a task) derived from WBAs completed by faculty members from multiple hospitals associated with a single, large, residency program at McMaster University, Canada. Narrative comments were vectorized to quantitative ratings using the bag-of-n-grams technique with 3 input types: unigram, bigrams, and trigrams. Supervised ML models using linear regression were trained with the quantitative ratings, performed binary classification, and output a prediction of whether a resident fell into the category of at risk or not at risk. Sensitivity, specificity, and accuracy metrics are reported.

**Results:**

The database comprised 7199 unique direct observation assessments, containing both narrative comments and a rating between 3 and 7 in imbalanced distribution (scores 3-5: 726 ratings; and scores 6-7: 4871 ratings). A total of 141 unique raters from 5 different hospitals and 45 unique residents participated over the course of 5 academic years. When comparing the 3 different input types for diagnosing if a trainee would be rated low (ie, 1-5) or high (ie, 6 or 7), our accuracy for trigrams was 87%, bigrams 86%, and unigrams 82%. We also found that all 3 input types had better prediction accuracy when using a bimodal cut (eg, lower or higher) compared with predicting performance along the full 7-point rating scale (50%-52%).

**Conclusions:**

The ML models can accurately identify underperforming residents via narrative comments provided for WBAs. The words generated in WBAs can be a worthy data set to augment human decisions for educators tasked with processing large volumes of narrative assessments.

## Introduction

Workplace-based assessments (WBAs) are a key source of data about the competence of health professions learners [1-9]. Even in the busiest of environments, clinical teachers engage in direct observation, feedback, and assessment of trainees [10]. The data gathered in these busy environments often consist of both quantitative (numerical scores, typically associated with a scoring rubric, such as an entrustment scale) and qualitative (free-form narrative comments) data [8].

Throughout training, WBA programs can acquire hundreds of data points about a single trainee, which translate into hundreds of scores and thousands of words [3]. While quantitative scores can be aggregated and analyzed using several statistical methods [11,12], qualitative data often require an educator (eg, program director [PD], competence committee [CC] member, learner supervisor) to internally organize and make meaning of the data. With the rapid and expansive generation of narrative comments typical of a robust and active WBA system, the cognitive task load can overwhelm administrators. This becomes even more problematic when aggregated narrative data inform progress decisions for advancement in training.

Machine learning (ML) algorithms and natural language processing (NLP) have been demonstrated in other industries and in general health care to provide near real-time data analysis of large complex qualitative data sets. Adopting these techniques in medical education may thus be useful [11,13,14]. Early work in using ML algorithms (MLAs) to enhance human review of the quantitative learner assessment data generated by WBAs has been reported [15]. However, as the systematic review by Dias et al [14] pointed out, much of the work reported to date is around feasibility.

For machine-assisted qualitative data aggregation or analysis, the field is sparse. Some qualitative data sets have shown potential in assisting faculty in identifying those trainees who are at risk [16]. Early research suggests that keyword-specific algorithms may assist human review of qualitative data from WBAs [17]. A recent systematic review of NLP within medical education showed that the majority of the research to date examines clinical notes generated by the trainee, rather than assessment data generated about the trainee [13].

Narrative data have been shown to be both reliable and useful [18-20]. Not only are written comments deemed reliable for third-party readers to interpret the progression of trainees [18], but also the learners often cite that they value these commentaries above scores or numbers [20,21]. Qualitative assessments contain both clarifying and qualifying data about the numerical scores. To be clear, qualitative data can still be biased [11,22]. Assessors have multiple competing interests, clouding their ability to focus on the assessment task [10]. Cognitive load for raters embedded in the workplace may also lead to limitations in the types of ratings they generate [23,24]. Moreover, individual faculty members may have social biases that manifest in their comments [25,26].

However, the operational challenge unique to qualitative data compared with quantitative data is the aggregation of multiple narrative assessments into a global judgment. The difficulty of this task requires approaches akin to the ones used with inductive research methods—multiple reviewers, all providing their own interpretations of the data, and working together to generate a common interpretation. To navigate this challenge, many assessment systems use CCs, which harness the power of group dynamics to arrive at decisions about complex data sets [27-30]. These committees function similar to promotion and tenure committees or juries, and are often charged with aggregating, reviewing, and interpreting multiple sources of data to arrive at decisions about trainee performance [31-33]. While this type of approach is a systematic and robust method, it neglects the operational challenges of navigating the large volume of data created by programmatic assessments using only human-based systems.

There is potential for harnessing NLP and ML for the purposes of automating the first analysis of narrative data from WBAs to generate red flags of underperforming learners. This automated, early warning system could facilitate the more nuanced human review of the same data of the identified individual, allowing educators to focus their efforts and offload the overwhelming cognitive load to more efficient NLP and MLA processes. While this technology has potential to support a potential automated process and to create an early warning system, this paper acts as proof of concept and presents an approach as to how we can utilize NLP and ML to automatize the assessment process to offload a system for busy clinical teachers. To do that, the MLA should be trained with existing data so that future WBA data can be analyzed automatically. The purpose of this study is to explore NLP and MLA applications for identifying trainees at risk using a large WBA narrative comment data set associated with numerical ratings.

## Methods

### Study Context

This study retrospectively analyzed all WBA data from September 2012 to July 2018 of emergency medicine residents completed by faculty members from a large, multihospital residency training program at McMaster University, Canada. This clinical setting has between 6 and 10 trainees within a 5-year specialist training program for emergency medicine; therefore, at any given time there are roughly 40 trainees in the program, but only 6 new trainees enter the system each year. The health system is also nontrainee dependent (ie, staffed entirely by attending physicians, who function independently without the assistance of trainees or midlevel providers), which means there are more than double the amount of faculty members than there are trainee physicians affiliated with the program. As such, while trainees always have a supervising attending physician who is their teacher/assessor for the shift [10], not all shifts staffed by an attending physician will have a trainee.

The McMaster Modular Assessment Program (McMAP) is a programmatic assessment system with 76 WBA instruments grouped by junior, intermediate, and senior level, and mapped to the CanMEDS (The Canadian Medical Education Directives for Specialists) roles [3]. We descriptively explain those competencies in Table 1 and provide the number of assessments for each competency. However, we focus on each WBA in our analysis. One WBA is completed during each emergency department shift. Free-form narrative comments and a behaviorally anchored 7-point score are captured for each WBA. A full WBA example form is presented in Multimedia Appendix 1.

### Analysis

A descriptive analysis of numerical scores and word frequencies was used to explore data and identify missing data. Demographics were analyzed using descriptive statistics in SPSS version 26 (IBM Inc.) [34]. Mean, SD, and frequencies were some of the descriptive statistics used. Missing data exploration was carried out on the data set to find ratings without comments and removed from the ML and NLP analyses. We used MATLAB R2019b and its libraries including “Statistics and Machine Learning Toolbox” and “Text Analytics Toolbox” to conduct analysis on MLA and NLP [35].

Two approaches were developed to stratify the data by quantitative rating. First, we used the 7-point scale ratings in the original form. To improve our ML models, we decided to collapse the ratings into a binary division. We chose this approach because many CCs promote a resident based on achieving a threshold (eg, a rating score 6.25 in our local setting for these WBAs, based on local standard-setting protocols) [3,36]. Thus, ratings from 1 to 5 were collapsed as a low score and ratings from 6 to 7 were collapsed as a high score.

### Natural Language Processing and Machine Learning Analysis

NLP and a supervised ML analysis were run sequentially to identify patterns and results. Our model takes the input of a written feedback review for a resident’s performance on a given day and tokenizes it to uni/bi/trigrams. Then, a linear regression ML model predicts the output for 2 different classifications: *at-risk* resident or *not-at-risk* resident.

### Step 1: Preprocessing

Preprocessing steps are described in Textbox 1.

**Preprocessing steps for narrative comments.**

1. Missing data: Assessment with no rating and comment was removed from machine learning algorithm analysis.
2. Tokenization: Each word was converted into a single-word format.
3. Part of speech: This function assigns a label to a word, such as verb, noun, proposition, number, punctuation.
4. Removal of stop words: To reduce noise in the data set, we removed stop words such as *a*, *and*, and *the*.
5. Lemmatization: Each word was converted into its root form (eg, *discharging* converted to *discharge*).
6. Removing punctuation: Punctuation was erased from the data set.
7. Removing infrequent words: Words with a frequency of 2 or fewer across the data set were removed.
8. Exclude empty assessment: Any blank narrative assessment fields were removed.

### Step 2: Vectoring

After preprocessing, we used bag-of-words vectorizing. We generated unigrams (single, decontextualized words), bigrams (adjacent word couplets), and trigrams (adjacent word triplets) for input into the ML models.

### Step 3: Machine Learning Analysis

#### Overview

Bag-of-words vectorizing for narrative data was used for the MLA stage. This technique takes each word within the comment and inputs each word into the MLA. Data were partitioned using a “holdout” technique with a 0.1 coefficient, meaning 10% of the data were randomly assigned with a nonstratified technique into a test data set, and the remaining data were selected for the training. ML analysis evaluated using tenfold cross-validation. More of the MLA explanation can be found in Multimedia Appendix 2.

#### Derivation Phase: Training of the Machine Learning Algorithm

The data were partitioned into a training and a testing data set. A supervised classification model, which used word frequency counts from the bag-of-words model as a predictor, was created and trained. The classification accuracy is the proportion of the labels that the model predicts correctly.

The supervised ML method used a linear learner model to train the data and to predict the test data set. Supervised learning can train a model when there are input data associated with a label as an outcome [37]. Our method is Error-Correcting Output Codes (ECOC), which uses K(K – 1)/2 binary support vector machine models, which means each classification group needs to be compared against the others. We did this by using the one-versus-one coding design, where K is the number of unique classification labels.

We trained the ECOC method composed of default classification models using the following parameters: *Learners* and *Linear.* The support vector machine used word frequency counts from the bag-of-words model as a predictor.

#### Validation Phase: Testing of the Machine Learning Algorithm

The last step was predicting the labels of the test data using the trained model and calculating the classification accuracy. Please see Multimedia Appendix 2 for further details on the training and testing phases.

### Ethical Consideration

The Hamilton Integrated Research Ethics Board granted ethics exemption for this study under Tri-Council Policy Statement 2 (TCPS2) as this was deemed a quality improvement initiative.

## Results

The initial database consisted of 7199 assessments, of which 5597 contained faculty comments for trainee performance. There were 141 unique raters from 5 different hospitals; 68% (n=96) of them were male. The database had a total of 45 unique residents; 56% (n=25) were male. Table 1 presents the overall demographics related to the assessments.

Rating distributions of the assessment ranged between 3 and 7. The frequencies for ratings 7, 6, 5, 4, and 3 were 2713/7199 (37.69%), 2158/7199 (29.98%), 635/7199 (8.82%), 79/7199 (1.10%), and 12/7199 (0.17%), respectively. We excluded a total of 1638 items because there were missing data (eg, the task rating did not have a meaningful comment associated or vice versa). The test set consisted of 484 high ratings and 72 low ratings.

In line with our previous work [15], we dichotomized our task rating scores: all scores of 5 and below were considered *at risk* and all scores of 6 and 7 were considered *not at risk.*

**Figure 1:**
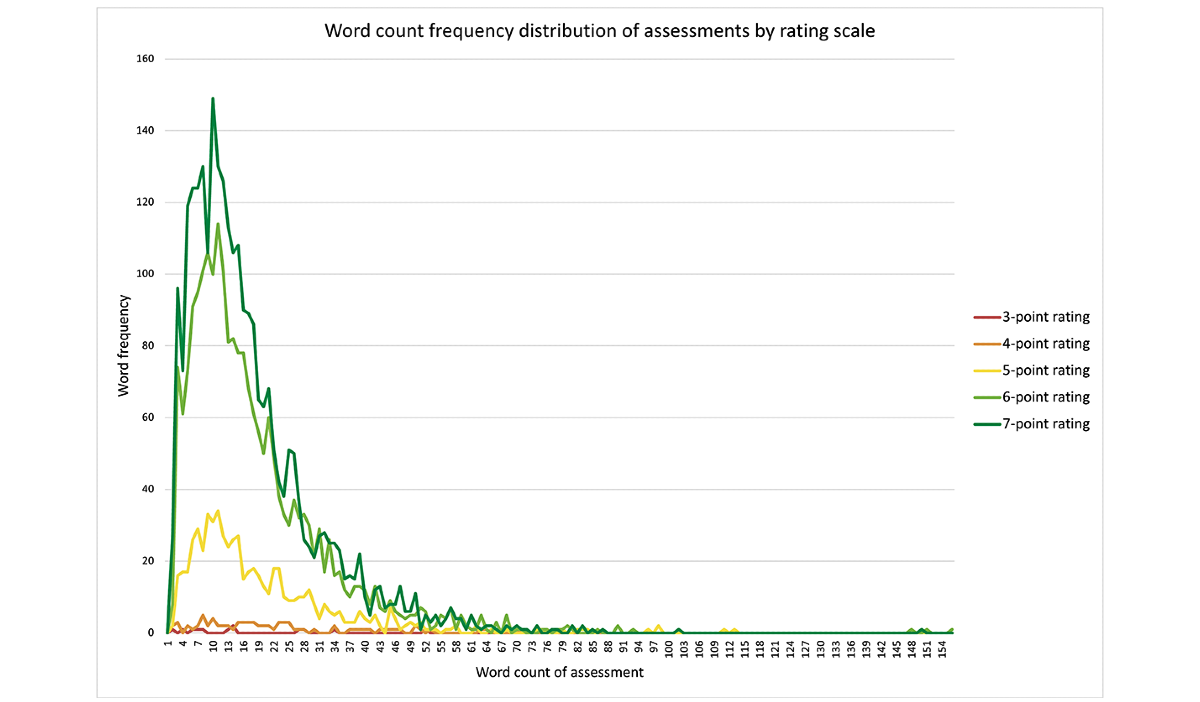
Distribution of word counts in comments within the assessments, split by the resultant scores.

There were 94,016 words in the narrative comments. Assessments ranged from 1 to 155 words with a mean of 16.91 (SD 13.8). Figure 1 shows the frequencies of word counts across assessments by rating scale. Each rating scale is represented with a color in Figure 1 and seemed to have a similar trend in each rating scale regardless of the number of ratings.

Multimedia Appendix 3 depicts word clouds with size-based weightings of unigrams, bigrams, and trigrams grouped by higher-score (6 or 7) and lower-score (≤5) associated phrases. Bigram analysis showed more promising weighted phrases such as *good approach* or *excellent management.* The trigram analysis highlights key phrases that allow a human reader to begin contextualizing the assessment such as *rapport patient family* or *excellent communication skill.* There are more diverse phrases in the trigrams associated with lower scores rather than those associated with lower scores.

Table 2 presents the MLA results for accurately identifying residents who were deemed *at risk.* Accuracy was higher using a binary division of the rating scale labeling. Trigrams provided the most accurate results. The MLA demonstrated excellent sensitivity for identifying residents who achieved competence (6 or 7 on the rating scale). Unigrams had the highest sensitivity rates. The specificity was poor. More details on the analysis output (ie, confusion matrix and area under the curve graphs) can be found in Multimedia Appendix 4.

**Table 1:** Assessment distribution across the data set (N=7199).

Distribution				Frequency, n (%)
**Postgraduate year**				
	1			1017 (14.13)
	2			3139 (43.60)
	3			405 (5.63)
	4			1585 (22.02)
	5			1053 (14.63)
**Categories of work-based assessments**				
	**Junior modules (PGY^a^1 and 2)**			
		Medical expert and scholar	1220 (16.95)	
		Advocacy and management	882 (12.25)	
		Communication and collaboration	1606 (22.31)	
		Professional and communicator	828 (11.50)	
		Pediatric emergency medicine	881 (12.24)	
	**Senior modules (PGY3-5)**			
		Leadership and team management	582 (8.08)	
		Quality decision making	805 (11.18)	
		Teaching and scholarship	395 (5.49)	
**Numerical rating scores**				
	Missing matching qualitative comment			1602 (22.25)
	3			12 (0.17)
	4			79 (1.10)
	5			635 (8.82)
	6			2158 (29.98)
	7			2713 (37.69)
**Binary classification**				
	Missing matching qualitative comment			1602 (22.25)
	1-5			726 (10.08)
	6-7			4871 (67.66)

^a^PGY: postgraduate year.

**Table 2:** Machine learning results on workplace-based assessments.

n-Gram input type	Sensitivity (%)		Specificity (%)		Accuracy of machine learning and natural language processing algorithms for diagnosing trainees at risk (%)	
	1-7 scale	Binary	1-7 scale	Binary	1-7 scale	Binary
Unigrams	82.0	92.2	5.1	13.9	51.8	82.0
Bigrams	81.1	87.3	4.1	21.4	50.4	85.6
Trigrams	79.6	87.0	Insufficient data to calculate	4.2	49.8	86.9

## Discussion

### Principal Findings

While NLP ML analyses have had many applications in health services (eg, interpreting large volumes of tweets or other data sets) [38,39], they are yet to be regularly used within the domain of aggregating and interpreting trainee-level data. This study demonstrates that an automated NLP ML analysis can identify resident performance that achieves competence on a direct-observation WBA using narrative comments.

While dichotomizing our 7-point assessment scale improved performance, the data set was not large enough to draw conclusions for specificity measures, due to a lack of true negatives within our trigram data. Specifically, our present ML model could not identify trainees who are failing with trigrams. The reason for low specificity was that our data had far fewer assessments on the lower end of the scale, especially for trigrams. While MLA can support the decision-making process, trainees who are at risk should be approached cautiously, triangulating data using human raters before decision making. However, the sensitivity of our algorithms suggests that we can harness the power of the NLP MLA to *rule out* the trainees who are not deemed at risk of meeting a performance standard. Human meta-raters could be most effectively deployed, then, to read those who have been flagged as possibly being at risk, and to make determinations of whether someone was truly at risk (eg, true positive) versus unduly flagged (eg, false positive). Moreover, McMAP is one of the first programmatic assessment systems in residency education [1,3,21]. It preceded a national shift to competency-based medical education by 6 years. There is no comparable pilot with similar accumulated data set yet since other programs began their system in 2017.

Based on this study, it is clear that larger data sets from amalgamated sources of common WBAs may hold the key to increasing the sampling (and therefore, the accuracy, sensitivity, and specificity) of our proposed algorithms. Early work within our specialty has shown that this may be possible [40], because all of specialist emergency medicine training have recently moved into a harmonized assessment system within Canada [41]. Finding ways to aggregate a nation’s worth of WBA across a specialty and multiple sites will undoubtedly afford us enough data to power NLP MLAs that can be helpful for faculty decision makers and decrease the workload introduced by robust WBA programs.

This automated process could obviate the need for a manual review of all qualitative phrases. While the specificity of the automated process is quite poor to identify residents who have achieved competence in the task, this process allows our CCs and PDs to continuously monitor their trainees’ performance. This will allow for an automated process to accurately identify trainees who may potentially require assistance or remediation. As our sensitivity ranged from 87% to 92.2%, we suggest that with higher stakes, summative decisions will still require human oversight and review to ensure that those who might be misclassified by the algorithm as requiring assistance (or needing more time) can be identified.

### Comparison With the Prior Work

An exploratory study of residents’ perception of WBA found that residents deemed feedback more valuable than numeric scores and acknowledged their skepticism on faculty comprehension of rating tasks [21]. Credibility is essential for feedback to be actionable. The factors that contribute to feedback credibility are the closeness of the relationship between supervisor and trainee, the consistency between the narrative and numeric score, and the quality of the narrative and a system that fosters a feedback culture [21,42]. This study demonstrates that ML and NLP can provide additional information on the evidence that supports results in WBA.

To complete a direct observation assessment, faculty undergo cognitive processes that involve observation, processing, and integration within the short time frame dedicated to the assessment [43,44]. When observing, the raters select the learner behaviors that are relevant to the assessment. These attributes may or may not be described in the narrative portion of the assessment. Processing involves the recollection of behaviors, matching behavior to a specific set or a subset of competencies, synthesizing the information collected, and integrating all the information into a narrative or numeric score [44]. Processing also responds to the individual conception of competency, context-specific settings, references to the highest and lowest performance witnessed by the rater, and emotions [43]. Intrarater reliability and consistency between narratives and numeric scores depend on the aforesaid cognitive process [45].

The interpretation of narrative comments is a complex task because words can be vague or have nonliteral meanings [18,46]. Raters and trainees decipher the alternative meanings of words using contextual information and experience. The precision of a narrative, the strength of the adjectives used, or specific references to competency domains are some of the elements to be considered when interpreting the hidden code [18,46]. As writing style differs between raters, the code is not universal and it can be mistakenly interpreted (eg, including areas of improvement in a narrative assessment might be considered positive or negative depending on the individual).

The traditional quantitative assessment paradigm leads learners and faculty to focus on numbers, and partially explains the complexity of the faculty task of “converting” or transferring their perception of competence into a 7-point scale. In fact, rater bias may be a result of the complexity of the unconscious action required to complete complex assessment tasks to assign scores to observations (very blunt, nonrich category) to a rater’s judgment.

While not realized within our study, NLP analyses have been shown to provide information on the quality, usefulness, and relevance of narrative assessment [47-49]. Moreover, it can generate insights about identity of raters, their cognitive process, potential biases, and personality traits. For instance, the use of determiners, prepositions, and pronouns have been identified as features for gender discrimination [50] and relevant linguistic differences have been found in narratives from male and female faculties [51]. While human meta-raters (ie,. those who read others’ comments) require more context about the feedback (eg, raters, audience, intent) [46], ML analysis can overcome the issues around context by increasing n-grams to match the scores based on qualitative data.

### Strengths and Limitations

This study is a worked example that is based on real trainee data and frontline faculty assessors in the context of WBA. With a diverse team of educators, computer scientists, and clinicians, we have been able to move the mark toward solving a problem that many medical educators are facing around qualitative comments.

However, our study has also some limitations. Residency training selects for highly qualified and high-performing learners. As a result, assessments have a positive yield that creates a right-skewed data distribution, where residents tend to have higher ratings rather than low ratings. Our data were no different. The range restriction of our data has impacted our results.

Our data set was not sufficient to create a validation set. In the future, with more data, we will likely move toward having an 80% derivation, with 10% testing and 10% validation profile for our data partitioning. We acknowledge that there are limitations of the output of the model, but unfortunately, we are limited to the results we could obtain with these data. This early work will allow us to approximate sample sizes and to further the field toward an eventuality where the technology we currently have can be properly harvested in this area. We anticipate, based on our early work, that we will need data sets that are amalgamated by a country’s worth of data to create the accuracy and precision required to truly make this a reality. With a larger data set we might have been able to complete more cross-validation procedures [52-56]. Human factors was another limitation in our study. Faculty members sometimes do not provide written comment with their ratings. Our study context is in an emergency department where there is not always time to provide any comment at all. We labeled them as missing in our study because we could not use them for NLP. Finally, our data set shows that greater pooling of data will be required by training programs (possibly across multiple centers or across a nation) to ensure that we have the depth of data to gain insights using NLP MLA technologies to advise CCs and PDs about trainees at risk. While there are some who might want to see a dichotomy between algorithms and humans, our team proposes that we should aspire for human-augmented decision making (eg, decision support), as opposed to assuming that MLAs might replace our training committees and faculty.

### Future Directions

Using N-grams with different scales showed a great promise on the retrospective data. These results beg for confirmation in a prospective study. While we used our WBA based on 2 different scales, we highly anticipate that this result will show a similar pattern in entrustment scales. Therefore, future research should focus on entrustment scales.

Next, greater data sets will be required to adequately harness the power of NLP and MLA technologies to assist faculty members or trainees in terms of decision making around academic or clinical progress. There have been some great strides recently made in creating amalgamated trainee assessment data sets for nationalized program evaluation [40], but full data pooling and sharing will be required to adequately generate the insights that are required using these technologies. Greater attention must be paid to create harmonized data standards and safe reporting protocols so that we can pool both quantitative and qualitative data required to capitalize on the technologies that currently exist, and are used regularly in other sectors.

Finally, NLP and ML must be tested against the current reference standard of CC-driven insights so that we can decide whether ML results are truly useful to augment faculty decision making and help improve the decision-making process.

### Conclusions

Our early data show promise that NLP with ML analysis of narrative assessment data may eventually serve as a decision-support system for CC, PDs, and other education decision makers. NLP and ML analyses have the potential to reduce the workload of large narrative data sets.

## Appendix

Multimedia Appendix 1 Sample McMAP task in knowledge translation.

Multimedia Appendix 2 Supplemental material for methods.

Multimedia Appendix 3 The different word cloud representations of the types of phrases found within comments associated with higher score (6 or 7 out of 7) and lower score comments (5 or less out of 7). While some similar word and phrases are found in the unigrams, bigrams, and trigrams, there is more homogeneity in the phrases within the higher score-associated trigrams and more diversity in the lower score-associated trigrams.

Multimedia Appendix 4 Confusion matrix for binary class.

## Abbreviations

CanMEDS: The Canadian Medical Education Directives for Specialists
CC: competence committee
ECOC: Error-Correcting Output Codes
McMAP: McMaster Modular Assessment Program
ML: machine learning
MLA: machine learning algorithm
NLP: natural language processing
PD: program director
WBA: workplace-based assessment
