# Supplementary material for: Harnessing Natural Language Processing to Support Decisions Around Workplace-Based Assessment: Machine Learning Study of Competency-Based Medical Education
Source: JMIR Med Educ. 2022 May 27;8(2):e30537. doi: 10.2196/30537 (PMC9187970; doi:10.2196/30537)
Supplement: Multimedia Appendix 1 [file mededu_v8i2e30537_app1.docx]

**Multimedia Appendix 1: Sample McMAP task in knowledge translation**

**Task: Knowledge Translation [1]**

Resident Name: Assessor Name: Date:

**Today’s focus is the Knowledge Translation**: Utilization and application of the Wells Score and PERC rule for Pulmonary Embolus.

For a given patient with Chest Pain, the resident will need to describe the application of the Well’s PE score, the PERC score, and the role of diagnostic tests in relation to the results of these scores.

| **Checklist** | **Done** | **Done but needs attention** | **Not done** | **N/A for case** |
| --- | --- | --- | --- | --- |
| Defines the PERC rule appropriately  - describes study inclusion criteria, exclusion criteria |  |  |  |  |
| Applies the PERC rule appropriately |  |  |  |  |
| Defines the Wells PE Score appropriately  - describes study inclusion criteria, exclusion criteria |  |  |  |  |
| Applies the Wells PE Score appropriately |  |  |  |  |
| Explains limitations of each of the respective scores / rules |  |  |  |  |
| Is able to describe the link between the Well’s PE score and the PERC rule. |  |  |  |  |
| Utilizes D-dimer appropriately in the context of these rules to guide management decisions (i.e. was the ordering of the d-dimer appropriate) |  |  |  |  |
| Interprets the D-dimer appropriately to guide management / treatment decisions |  |  |  |  |

| **Rate this task Circle the number that that best describes level of proficiency** | | | | | | |
| --- | --- | --- | --- | --- | --- | --- |
| **1 Needs assistance** | **2** | **3** | **4** | **5** | **6** | **7 Ready for the next level** |
| *Displays the following behaviours:*  • Not able to define the rule(s) or literature  • cannot apply it to the patient. |  | • Able to define the rule  • is not clear about application of the rule(s) or literature to the patient. |  | • Able to define the rule, and apply the rule(s) the patient  • Is unclear about limitations of the rule(s) or literature  • cannot describe the subsequent  management steps. |  | *Displays all of the following behaviours:*  • Is able to define the rule(s) or literature, can apply it to assist with decision making.  • Is able to articulate the limitations and describe the subsequent management steps. |

**The Evidence:** Please provide an example with an explanation that supports your rating (MANDATORY)

**The next step:** Based on the above evidence, please give one specific suggestion (Education Prescription) for the resident to attempt during his/her next shift. (You do not need to record this).

1. Chan T, Sherbino J. *McMaster Modular Assessment Program Junior Edition*. Academic Life in Emergency Medicine; 2015. doi:10.13140/RG.2.1.3452.8168
