# Supplementary material for: Harnessing Natural Language Processing to Support Decisions Around Workplace-Based Assessment: Machine Learning Study of Competency-Based Medical Education
Source: JMIR Med Educ. 2022 May 27;8(2):e30537. doi: 10.2196/30537 (PMC9187970; doi:10.2196/30537)
Supplement: Multimedia Appendix 2 [file mededu_v8i2e30537_app2.docx]

**Multimedia Appendix 2: Supplemental material for methods.**

ML analysis can provide details of the test results if the ML model can correctly label and identify given input [1,2]. Confusion matrix can provide the results of test statistics which can be reported as accuracy measures. Sensitivity and specificity are two statistics that can be used to identify labeled class and output class in ML analysis [3]. Sensitivity and specificity are also highly used terms in medical diagnosis.[1](https://www.zotero.org/google-docs/?79icjG) Altman and Bland define sensitivity and specificity as the “true positives” and “true negatives” rate, respectively, which a test can correctly identify [4]. Thus, we used these terms in our study to correctly classify assessments which can fall under different scales for the residents’ performances. Specifically, sensitivity metric means the true positive proportion that ML analysis can identify residents' performance based on the task comment. On the other hand, specificity is the true negative that ML analysis can highlight the residents who are failing in the task. As a last performance metric, we used accuracy which is the measure of correctly labeled ratio across the dataset. Therefore, higher accuracy means that we are able to label the trainees correctly. Accuracy is the metric for the combination of sensitivity and specificity. Accuracy can give overall meaning across the dataset by determining true positive and true negatives within the data [5]. We used accuracy measures to determine overall performance of the ML model in the study. Using accuracy metric, we proposed that the ML model was successful in flagging trainees performance across the data set. Table S1 provides details about calculation formula and the use case in the study.

True positive (TP) = the number of assessments correctly identified as rated ready for progression

False positive (FP) = the number of assessments incorrectly identified as rated ready for progression

True negative (TN) = the number of assessments correctly identified as rated not ready for progression

False negative (FN) = the number of assessments incorrectly identified as rated not ready for progression

**Table S1: Definitions and explanations of machine Learning metrics used in the study**

| **Metric** | **Definition** | **Formula** | **Use in the study** |
| --- | --- | --- | --- |
| accuracy | Ratio of correctly predicting cases out of the whole cases. | $\frac{TP+TN}{TP+TN+FP+FN}$ | Ratio of the correctly labeled assessments to the original rating which were given by faculty members. How many assessments did ML correctly label out of all assessments? |
| sensitivity | Ratio of correctly predicting positive cases out of the whole positive cases. | $\frac{TP}{TP+FN}$ | Ratio of the correctly labeled ready for progression assessments to the originally rated by the faculty members. Of all the trainees who are ready for progression, how many did ML correctly predict? |
| specificity | Ratio of correctly predicting negative cases out of the whole negative cases. | $\frac{TN}{TN+FP}$ | Ratio of the correctly labeled not ready for progression assessments to the originally rated by the faculty members. Of all the trainees who are not ready for progression, how many did ML correctly predict? |

**References**

1. Liu Y, Chen P-HC, Krause J, Peng L. How to Read Articles That Use Machine Learning: Users’ Guides to the Medical Literature. [*JAMA*. 2019;322(18):1806. doi:10.1001/jama.2019.16489](https://www.zotero.org/google-docs/?6yAwSn)

2. Doshi-Velez F, Perlis RH. Evaluating Machine Learning Articles. [*JAMA*. 2019;322(18):1777. doi:10.1001/jama.2019.17304](https://www.zotero.org/google-docs/?6yAwSn)

3. Ariaeinejad A., Patel R., Chan T.M., Samavi R. Using machine learning algorithms for predicting future performance of emergency medicine residents. [*Can J Emerg Med*. 2017;19(Supplement 1):S88. doi:10.1017/cem.2017.233](https://www.zotero.org/google-docs/?6yAwSn)

4. Altman DG, Bland JM. Diagnostic tests. 1: Sensitivity and specificity. [*BMJ*. 1994;308(6943):1552.](https://www.zotero.org/google-docs/?6yAwSn)

5. Baratloo A, Hosseini M, Negida A, El Ashal G. Part 1: Simple Definition and Calculation of Accuracy, Sensitivity and Specificity. [*Emergency*. 2015;3(2):48-49.](https://www.zotero.org/google-docs/?6yAwSn)
