# Supplementary figures and images for: Harnessing Natural Language Processing to Support Decisions Around Workplace-Based Assessment: Machine Learning Study of Competency-Based Medical Education
Source: JMIR Med Educ. 2022 May 27;8(2):e30537. doi: 10.2196/30537 (PMC9187970; doi:10.2196/30537)

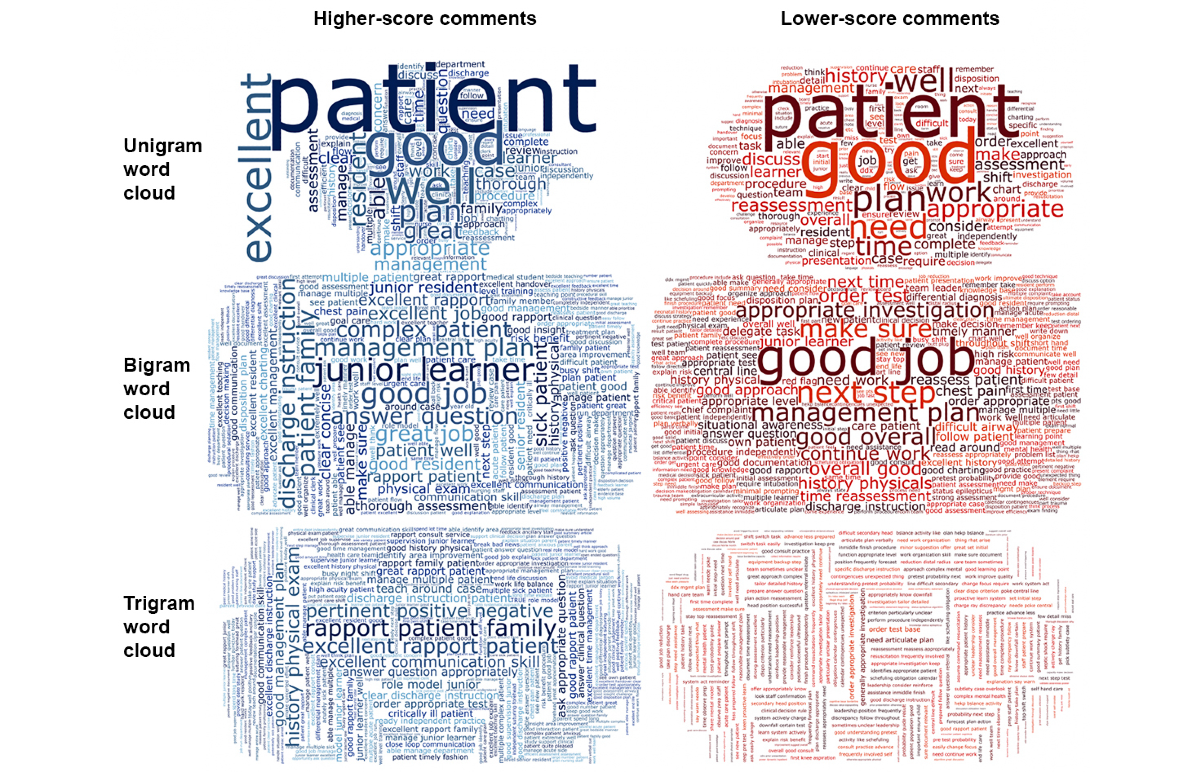

Supplement: Multimedia Appendix 3 [file mededu_v8i2e30537_app3.png]
