# Supplementary material for: Harnessing Natural Language Processing to Support Decisions Around Workplace-Based Assessment: Machine Learning Study of Competency-Based Medical Education
Source: JMIR Med Educ. 2022 May 27;8(2):e30537. doi: 10.2196/30537 (PMC9187970; doi:10.2196/30537)
Supplement: Multimedia Appendix 4 [file mededu_v8i2e30537_app4.docx]

**Multimedia Appendix 3: Confusion matrix for binary class.**


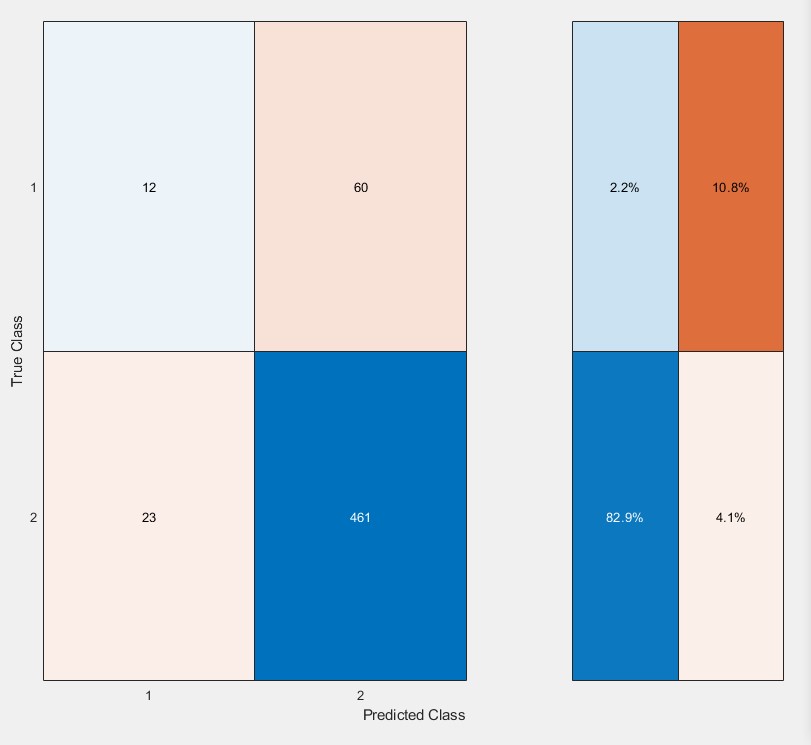


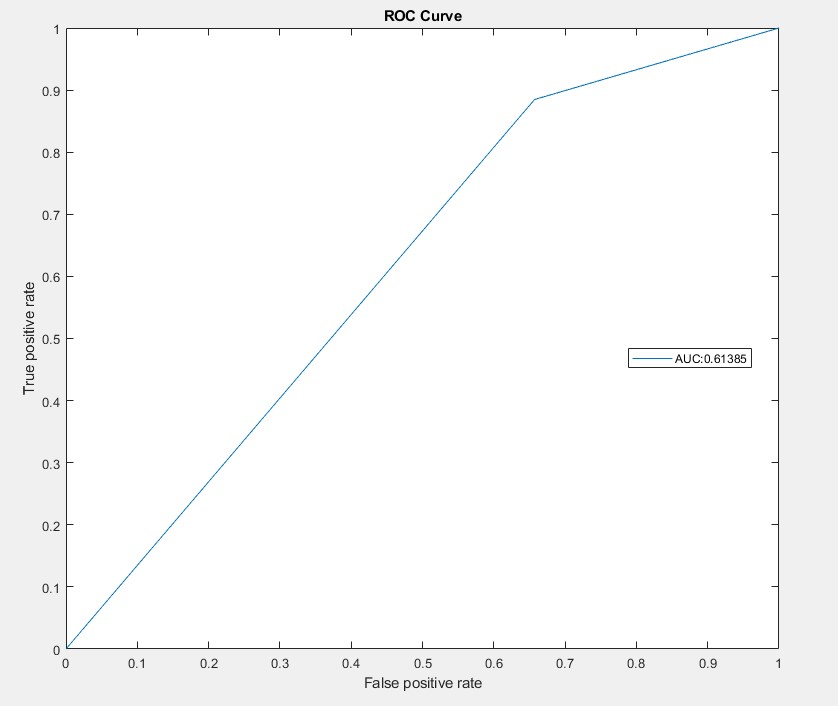


ROC Curve for Binary Class


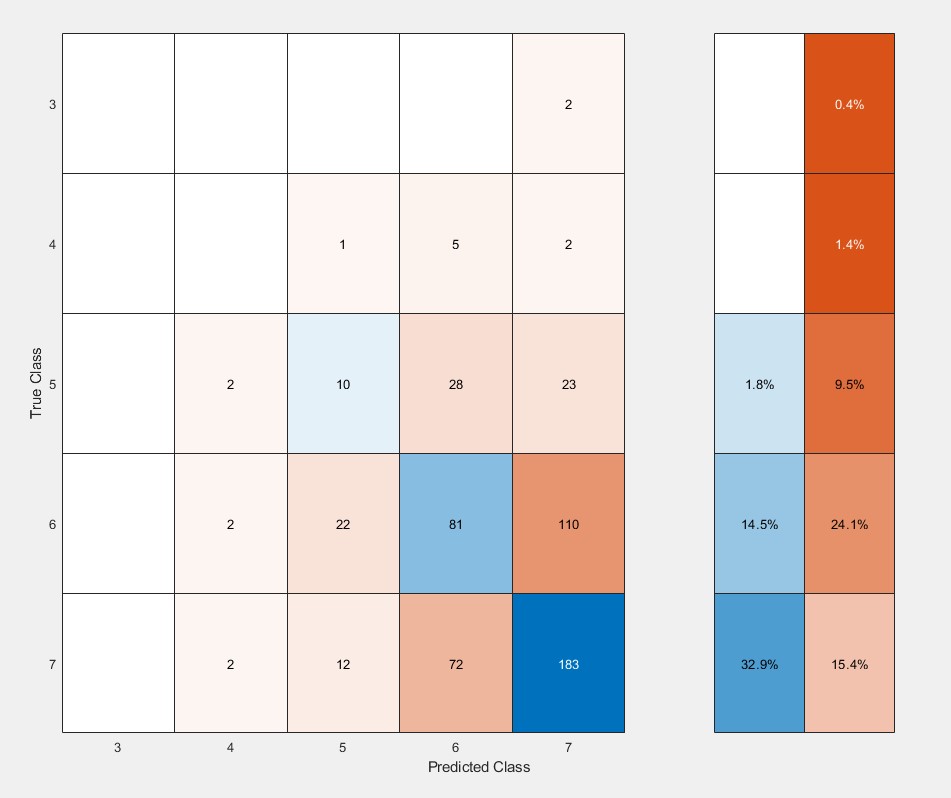


Confusion matrix for original scale (1-7).


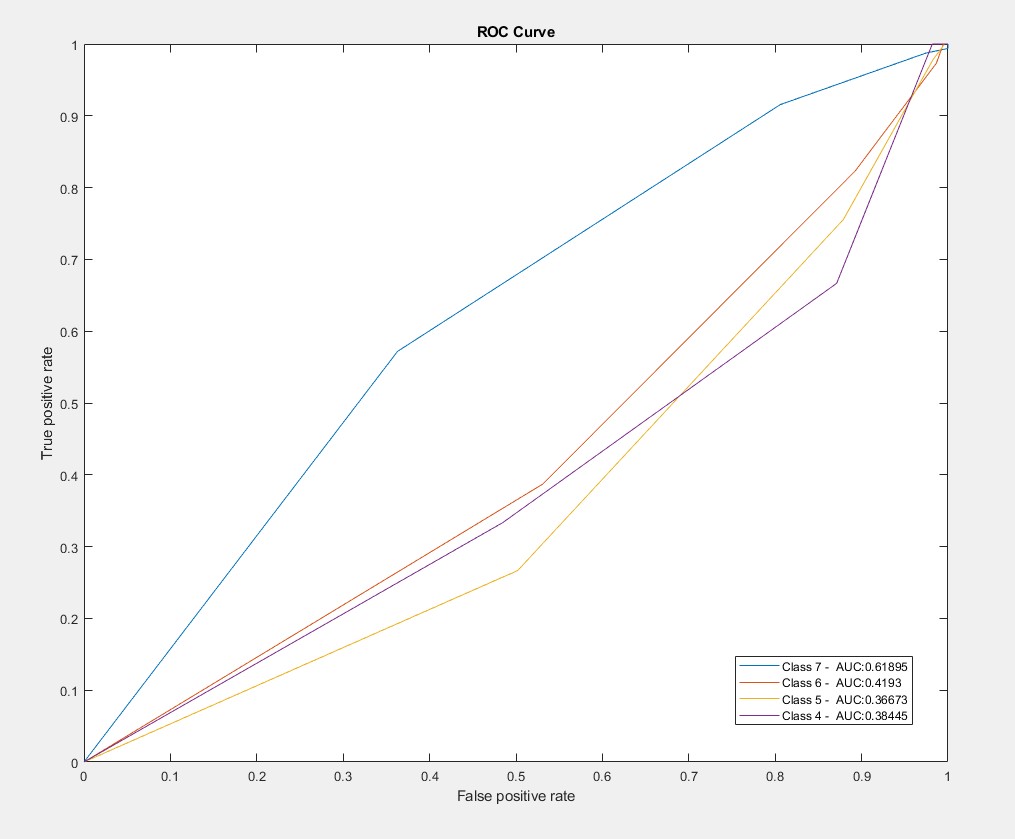


ROC Curve for Original Scale (1-7)
